# *Trichoderma*-Activated Granulated Digestate as an Alternative to Chemical Fertilization: Effects on Tomato Yield and Quality, and Soil Rhizospheric Communities

**DOI:** 10.1021/acsagscitech.5c00338

**Published:** 2025-11-21

**Authors:** Tihomir Petrov Petrov, Mattia Rizzetto, Elisa Clagnan, Marta Dell’Orto, Patrizia De Nisi, Giuliana D’Imporzano, Marco Ovani, Marco Pierpaolo Pina, Roberto Kron-Morelli, Fabrtizio Adani

**Affiliations:** † Gruppo Ricicla laboratories., Dipartimento di Scienze Agrarie e Ambientali − Produzione, Territorio, Agroenergia (DiSAA), Università degli studi di Milano, Via Celoria 2, 20133 Milano, Italy; ‡ Wrote Srl, Via Plinio, 1, 20124 Milano, Metropolitan City of Milan, Italy; § Agrifutur Srl, Via Campagnole 8, 25020 Alfanello, Province of Brescia, Italy

**Keywords:** *Trichoderma*, biobased fertilizers, biostimulation, microbiological activation, digestate, yield improvement

## Abstract

The use of synthetic fertilizers is always more economically and environmentally unsustainable. It is necessary to improve current agricultural practices. Bioactivated fertilizers are a promising solution to enhance digestate solid fraction’s fertilizing properties with an ad hoc microbial consortium and reach yields comparable to chemical fertilization (CF), thus combining circular economy with an upgraded organic agriculture. This study designed a new granulated formulation, obtained using a vacuum drying process at the industrial level, for an improved *Trichoderma*-activated digestate’s solid fraction. This granulation aimed to improve both management operations and *Trichoderma* activity. After a greenhouse experimentation, yields obtained from the activated digestate (56 ± 7 g FW plant^–1^) were similar to the one obtained with CF (62 ± 9 g FW plant^–1^). Additionally, the bioactivated digestate gave yield production that were 21–30% higher yield than that of digestate alone. Microbial activation further led to higher nutritional values with an increment in the lycopene content between 8.8% and 15.8%. A metagenomic analysis further highlighted the persistence of *Trichoderma* in the tomato rhizosphere and its ability to establish positive interactions with other beneficial rhizospheric microorganisms. Activated digestate showed its potential to substitute CF, while granulation resulted in a functional formulation to convey this product.

## Graphical abstract

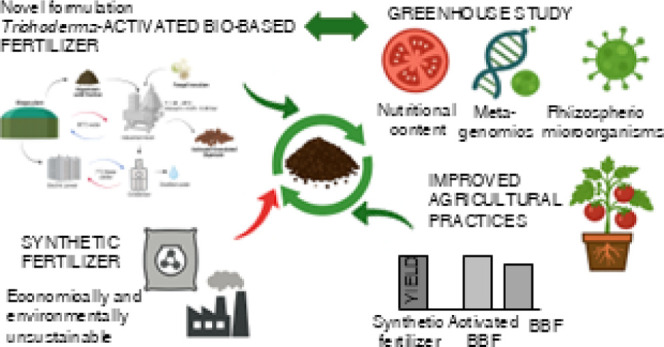

## 1 Introduction

Global population is expected to reach 9.9 billion people by 2050; therefore, there is the need to increase current food production by 70%.^1–3^ To improve yields, the agricultural sector has been applying intensive agronomical practices such as a wide use of chemical fertilizers and agrochemicals and continuous cultivation with no soil rest.^4–6^ These practices are known to have negative environmental impacts and translate into loss of soil organic carbon,^7^ water contamination,^8^ and biodiversity^6^ and the increase of greenhouse gases (GHGs) emissions.^9^ All these factors contribute to the unsustainability of the agricultural sector, and, therefore, there is a society need to increase crop yield, decrease GHGs emissions, and maintain crop and environmental quality.

A possible solution to reach sustainability consists in reducing the use of chemical fertilizers shifting to organic fertilizers.^10^ Nevertheless, it is well-known that the efficiency of organic fertilizers to supply nutrients to crops is limited in comparison to mineral fertilizers.^10,11^ Within organic fertilizers, nutrients (nitrogen (N) and phosphorus (P)) are mainly under organic form and therefore they need to be mineralized to reach an available form for plant uptake.^12^ Additionally, due to the low nutrient ready availability of organic fertilizers, plants’ nutrient requirements are often not met when needed, consequently these nutrients might become available later with harmful effects on the environment due to possible N leaching and/or P accumulation in soil.^13^

Recently, Clagnan et al.^14^ showed that bioactivation, i.e., the addition of beneficial microorganisms to organic matrices to improve the fertilizing properties of these organic fertilizers (bioactivated fertilizers), can substitute mineral fertilization with a comparable impact on crop yields. Although the microbial consortia used contained multiple microorganisms, *Trichoderma* spp. emerged as the leading actor in obtaining the positive results of the study.^14^ *Trichoderma* is well-known for its positive effects that are related to the production of biostimulative molecules,^15–17^ antistress properties,^18,19^ and a high ability to degrade organic matter, thus improving nutrient availability.^20–22^ Bioactivated fertilizers can be therefore considered as composed of an organic matrix able to host and support the growth of plant beneficial microorganisms.

Anaerobic digestion is a well-known bioprocess able to degrade and stabilize the organic matter contained in a biomass to achieve two main final products: biogas and a nutrient-rich digestate.^23,24^ Digestate is mainly composed of a well biologically stabilized organic matter that is useful as organic amendment to improve soil fertility and contribute to plant nutrition after the mineralization of its nutrients.^24,25^ Additionally, digestate contains also readily available nutrients under mineral forms (ammonia and phosphate), and meso- and micronutrients that can be used for crop growth.^26,27^ Furthermore, digestate is also characterized by biostimulant activity such as well documented in the literature.^28,29^

A simple solid–liquid separation of the digestate allows the production of two fractions with different properties: a liquid fraction that is characterized by a high nutrient concentration under mineral and water-soluble form that is currently used as a valid alternative to mineral fertilizers;^27^ and a solid fraction which is characterized by a high organic fraction and nutrients mostly under organic form, presenting optimal amendment properties but low nutrient availability.^25,27^ Digestates’ solid fraction can be, however, used as organic fertilizers for crops that do not require a large amount of nutrients in short periods. For crops requiring large amount of nutrients, solid digestate needs to be dosed in large quantity, creating environmental problems leading to temporary anoxic condition, nitrate leaching, P accumulation, and both ammonia and N_2_O emission, that are in contrast with a sustainable agriculture.^13^

As previously stated, the bioactivation of organic fertilizers improved nutrient availability and crops growth making organic fertilizers similar to mineral fertilizers.^14^ Therefore, the bioactivation of the solid fraction of digestate can open the way to a novel class of fertilizers, i.e., bioactivated organic fertilizers, leading, also, to new interesting commercial opportunities, as the organic fertilizers market is expected to the growth of about 10% y^–1^ from now to 2030.^30^ New formulations (such as granulation) are also needed as field application of bioactivation organic fertilizers is still hindered due to a lack of specialized equipment to facilitate their management.^31^

This study was aimed at developing and testing a bioactivated granulated fertilizer achieved by using an innovative process at Technology Readiness Level (TRL) of 8, able to dry the solid fraction of digestate together with *Trichoderma* spp. at low temperature and pressure, preserving the microorganism’s vitality. The granulation of the digestate with the microorganism creates a favorable niche for *Trichoderma* spp. that will benefit from the organic matter and nutrients when spread on the soil after the microorganism will reactivate thanks to the presence of water.

## 2 Materials and Methods

### 2.1 Fungal Inoculum and Activated Granulated Fertilizer

The fungal inoculum (FI) used was provided by Agrifutur Srl (Alfianello, BS, Italy) as a lyophilized powder consisting of a mix of two fungal strains, *Trichoderma harzianum* and *Trichoderma longibrachiatum* (2.5× 10^8^ spores).

The FI was supplied with two different formulations: (i) as a water suspension and (ii) a microbially activated granulated digestate (AGD). The microbial consortium was provided at 2.5 kg ha^–1^.

The activated granulated fertilizer (commercial name: Bio Boost) was produced by Wrote Srl (Milan, Italy) using a patented drying process (Patent number: 102022000021792; Title: Nuovi Biostimolanti; Ministero delle Imprese e del Made in Italy, 21/10/22) (Figure 1, Table S1, see Section 2.4). Briefly, a solid fraction of a bovine digestate (D) collected in the Lombardy region (Table S1, see Section 2.4) was mixed with the FI (18.5 g FI kg^–1^ DW digestate). The mix was then inserted into a dryer (10 m^3^ of capacity). The drying process was carried out at 40–46 °C, under vacuum (0.05–0.30 bar), and with continuous stirring for 4–6 h. Heat was provided by a biogas plant present on site treating slurry and energy crops. The produced AGD was stable and ready to use. After spreading, granules were rehydrated spontaneously, thanks to soil moisture, and consequently, microorganisms within the granules were reactivated and became viable.

**Figure 1:**
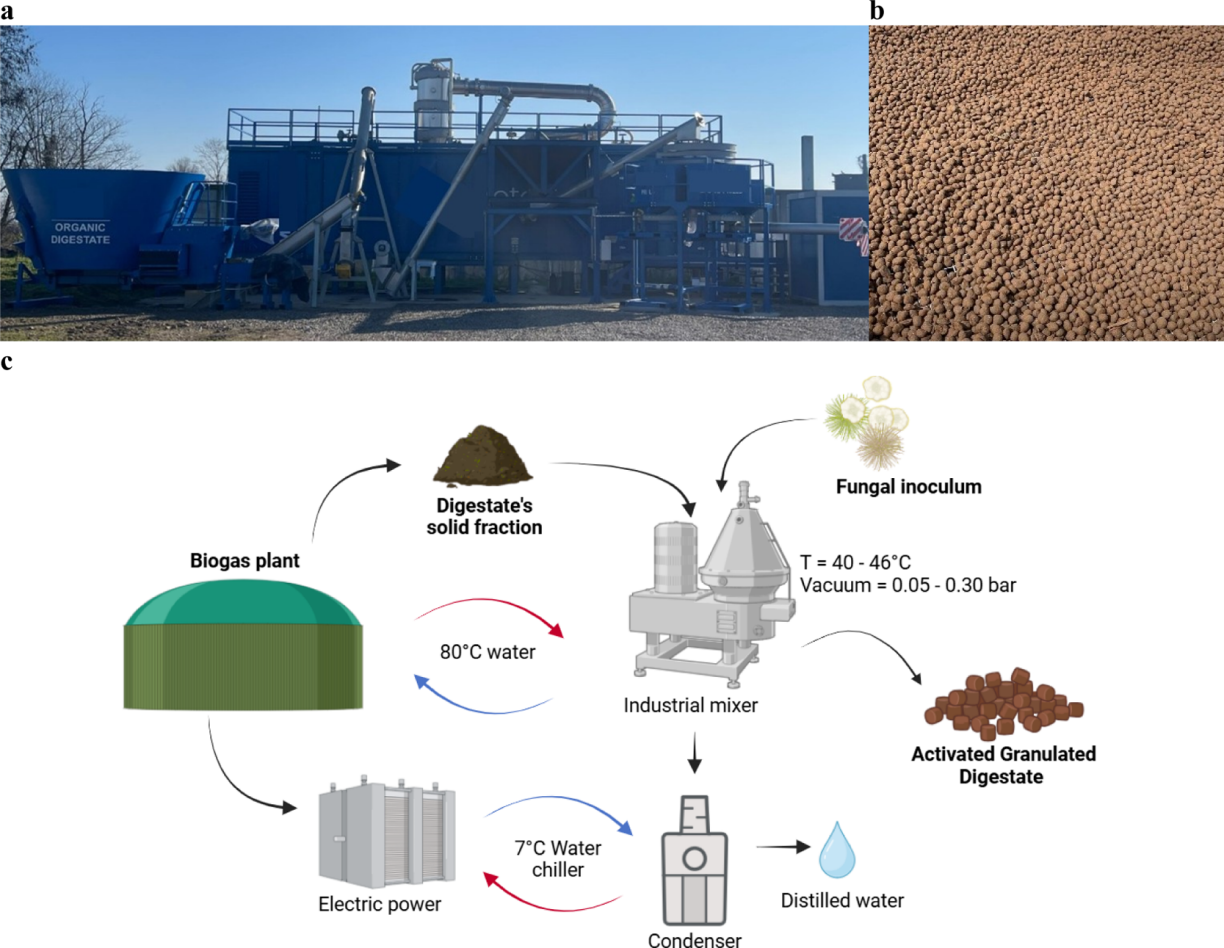
Pictures of the pilot plant (a) and of the produced AGD (b); and schematical representation of the production process (c).

### 2.2 Experimental Design

The experimental trial was carried out under greenhouse conditions for 7 months (temperature 25 °C, photoperiod 16 h light and 8 h dark per day) at the University of Milan (Italy).

Tomato (*Solanum lycopersicum* L. var. *lilliput* V.F.N. hybrid F.1) seeds (∼300) were sown and grown until the formation of the fourth leaf. Plants were selected for homogeneity by height, color, and number of leaves. Homogeneous plants (*n* = 25) were then transplanted into pots (1 plant per pot). Each pot contained 1.7 kg of a loamy soil collected at Sant’Angelo Lodigiano (LO, Italy; N 45.22, E 9.39) and homogenized by hand (Table S2; see Section 2.4).

Five treatments were considered: (i) no fertilization (NF); (ii) chemical fertilization (CF); (iii) digestate solid fraction fertilization (DF); (iv) digestate solid fraction fertilization with the addition of the microbially AGD (DF + AGD); and (v) digestate solid fraction fertilization with the addition of the microbial consortium as a water suspension (DF + FI). The NF treatment was used as a negative control of all fertilization treatments against the soil background; the CF treatment was used as control to assess and compare the effect of the synthetic fertilizer against all the organic fertilization treatments, while the DF treatment was used as a nonactivated control against both DF + AGD and DF + FI as the granules were composed of the same digestate of the DF treatments and the digestate content was dosed equally among treatments. A digestate sterile control was not added, to assess the specific effects of the microbial community of the digestate against the effect of *Trichoderma* as the goal of the study was not a mechanistic characterization of the activity of *Trichoderma*, but to achieve an improved effect by using *Trichoderma* in combination with the known biostimulant activities of the digestate itself derived by both its microbial community and biostimulant molecules in a real-life scenario. Additionally, an extra sterilization step (e.g., temperature or ultraviolet) could have led to a modification of its chemistry, organic compounds, and nutrient availability,^32^ therefore invalidating its comparability with nonsterile digestate.

The fertilization scheme is provided in Table S3. Briefly, fertilization was provided for all treatments (NF excluded) by dosing: 130 kg ha^–1^ of N, 100 kg ha^–1^ of P_2_O_5_, and 185 kg ha^–1^ K_2_O, according to tomato requirements. CF was provided using urea, Ca­(H_2_PO_4_)_2_, and K_2_SO_4_ as powders before transplantation of the tomato plants. The digestate solid fraction was provided before the transplant of the tomato plants according to its N content with the addition of K_2_SO_4_.

Pots were maintained at 65% of the maximum water holding capacity throughout the whole experiment.

### 2.3 Soils and Digestates Characterization

Before the experimental trial, soil (topsoil 0–20 cm) was collected into sterile bags and brought to the laboratory and characterized according to standard procedures following Zilio et al.^33^

Both D and AGD were characterized for dry weight (DW), volatile solids (VS), pH, total organic carbon content (TOC), total nitrogen, ammonia (N–NH_4_), P and potassium (K), and micro- and macro-nutrients, as previously reported by Clagnan et al.^14^

Soil and digestate characteristics can be found in Tables S1 and S2, respectively.

### 2.4 Crop Yields, Quality, and Nutritional Parameters

Tomato fruits were collected throughout the entire experimental period once the fruit reached full ripeness. To assess the ripeness, a colorimeter (Chroma Meter CR-410, Konica-Minolta, Milan, Italy) was used. Fruits were collected in a redness range (a*) of 25 ± 2. Tomatoes were stored at −20 °C for successive characterization. Tomato yield productions were measured by determining both fresh weight (FW) and DW (drying at 45 °C in a ventilated oven until constant weight).

Tomatoes (5 randomly selected tomatoes per treatment), thawed at 4 °C, were blended. The puree was then used to assess total soluble sugars (TSS) by using a digital refractometer (Atogo mod. N1, Tokyo, Japan), pH by using a pH meter (Eutech PC 2700, accuracy: ±0.002 pH ± 1 LSD), and lycopene content.^34^

Dried tomato fruits (45 °C until constant weight) were blended to a powder to determine the N content and quantify micro- and macro-elements.^14^

### 2.5 Statistics

All statistical analyses were performed by R studio (version 4.4.3). The data were processed by ANOVA, followed by mean separation through the Duncan test (*p*-value <0.05).

### 2.6 16S and ITS rRNA Genes Sequencing

Samples for NGS analyses were collected both before (T0) (D; AGD; FI and soil) and after (T1) of the experimental trial (rhizosphere soil for NF, CF, DF, DF + FI, and DF + AGD). For rhizospheric soil, roots were vigorously shaken, and the soil, which remained attached to the root system, was collected. Samples were further homogenized with sterile 2 mm sieves and stored at −80 °C prior to DNA extraction.

DNA was extracted in triplicate using a DNeasy PowerSoil Pro Kit (Qiagen, Germany) according to manufacturer’s instructions. The DNA yield was determined by a qubit (Thermo Fisher Scientific, USA), purity by a Nanodrop 1000 spectrophotometer (Thermo Fisher Scientific, USA), and integrity by gel electrophoresis on 1% (w/v) 1 × TAE agarose gels. DNA samples were stored at −80 °C prior to analyses.

DNA samples were shipped to Novogene (Cambridge, UK) and sequenced on an Illumina NovaSeq PE250 platform. The NGS was performed in duplicate targeting the V3 and V4 regions of the bacterial 16S rRNA gene using the 341F (CCTAYGGGRBGCASCAG) and 806R (GGACTACNNGGGTATCTAAT) primers.^35^ For the ITS1 regions of the fungal communities, primers were ITS 1f-F (CTTGGTCATTTAGAGGAAGTAA) and ITS1-1F-R (GCTGCGTTCTTCATCGATGC).^36^

The nucleotide sequences generated and analyzed are available at the NCBI SRA repository (BioProject accession number: PRJNA1256514).

Reads’ quality was checked through FastQC software. Reads were further analyzed using DADA2 for R as per https://benjjneb.github.io/dada2/tutorial.html. For taxonomic assignments, the SILVA and UNITE databases were used.

All statistical analyses were performed in R studio (version 4.4.3). Briefly, the package vegan was mainly used, while taxonomic summaries were performed using the phyloseq package. The Shapiro–Wilk test was used to assess normality, differences among samples of normally distributed data were tested by one-way analysis of variance (ANOVA), followed by Tukey’s post hoc test, while non-normal data were analyzed through a nonparametric Kruskal–Wallis test followed by Dunn’s test for multiple comparisons. For pairwise comparison, *t*-test and Wilcoxon signed-rank tests were used for normal and non-normal data, respectively. Beta diversity was tested through a PCoA with the microeco package, and then results were confirmed through a PERMANOVA test.

## 3 Results and Discussion

### 3.1 Yield and Nutritional Results

Yield results obtained for each treatment are reported in Table 1. As expected, the NF treatment produced the lowest yield among all treatments (3.3 ± 0.5 g of DW plant^–1^). Treatment DF (3.9 ± 1 g DW plant^–1^) showed significantly higher values than NF, most likely due to the nutrients provided by the digestate.^37^ The yield of treatment DF was lower than those of CF (5.1 ± 0.8 g of DW plant^–1^) and both bioactivated treatments (DF + FI: 4.6 ± 0.8 g of DW plant^–1^; DF + AGD: 5.6 ± 0.6 g of DW plant^–1^) since nutrients in digestate are mostly present in a low-available (organic) form and require mineralization before plant uptake. The bioactivation of digestate, i.e., DF + FI and DF + AGD treatments, allowed to achieve a crop yield not significantly different from that of the CF treatment, even though the digestate was the only source of nutrient as in treatment DF. As previously reported, the bioactivation of digestates generally leads to higher crop production than when using solely digestate.^14,21^ In particular, the bioactivated digestate gave yield production that was 21% and 30% higher (*P* < 0.05) than that of DF, for DF + FI and DF + AGD, respectively, suggesting the efficacy of the bioactivated digestate.

**Table 1:** Tomato Yield, Nutritional Characteristics

	NF^a^	CF	DF	DF + FI	DF + AGD
yield production (g FW plant–1)*	36 ± 5^b^c^c^	62 ± 9a	43 ± 11bc	52± 8ab	56 ± 7a
yield production (g DW plant–1)*	3.3 ± 0.5c	5.1 ± 0.8a	3.9 ± 1bc	4.6 ± 0.8ab	5.6 ± 0.6a
lycopene (mg kg–1 FW)	56.7 ± 5e	84.2 ±0c	77.4 ± 0d	97.4 ± 1a	91.6 ± 4b
total soluble sugar (°Bx)	5.5 ± 0.1c	5.3 ± 0.0c	6.4 ± 0.1b	6.8 ± 0.1a	6.5 ± 0.1b
pH (pH unit)	4 ± 0a	4 ± 0a	4 ± 0.a	4 ± 0a	4 ± 0a
tomato dry weight (% FW)	9.1 ± 1a	8.3 ± 1a	9.1 ± 0a	9.0 ± 1a	9.6 ± 0a
N (g kg^–1^ DW)	12.1 ± 0.1c	17.3 ± 0.1a	13.7 ± 0.9b	13.3 ± 0.0bc	13.8 ± 0.8b
P (g kg^–1^ DW)	4,820 ± 223a	4,576 ± 465a	4,491 ± 299a	4,537 ± 553a	4,357 ± 39a
K (g kg^–1^ DW)	37,263 ± 1,361ab	37,635± 78a	34,307 ± 1,795c	34,413 ± 558c	34,992 ± 1,833bc
Ca (μg g^–1^ DW)	170 ± 11c	220 ± 4a	209 ± 25ab	186 ± 8bc	167 ± 12c
Mg (μg g^–1^ DW)	1,906 ± 136a	1,852 ± 158a	1,754 ± 139a	1774 ± 261a	1732 ± 30a
Na (μg g^–1^ DW)	438 ± 10bc	441 ± 10abc	460 ± 27ab	412 ± 1c	482 ± 38a
Fe (μg g^–1^ DW)	33.2 ± 5a	35.0 ± 5a	27.8 ± 4a	28.5 ± 6a	31.3 ± 0a
Cu (μg g^–1^ DW)	7.0 ± 0.7a	7.6 ± 0.9a	6.2 ± 0.3ab	6.2 ± 1.6ab	5.2 ± 0.2b

a NF: no fertilization; CF: chemical fertilization; DF: digestate; DF + FI: digestate plus the microbial consortium; DF + AGD: digestate plus the AGD.

b Average and standard deviation, *n* = 3 (*n = 5).

c Along each line, values associated to different letters significantly differ according to the Duncan test (*P* ≤ 0.05).

Digestate has proven to have promoting effects on tomato growth and yield further improving photosynthesis due to its biostimulant activities and nutrient contents.^38^ Similarly, the presence of *Trichoderma* can further amplify these effects, leading to a good performance of the bioactivated fertilizers confirming previous findings by Clagnan et al.^14^ *Trichoderma* are facultative plant symbionts applied successfully in agriculture; they can interact with plants’ roots through a plethora of signaling molecules (e.g., cell-wall degrading enzymes, nonenzymatic secreted proteins, and specialized metabolites such as volatile organic compounds, plant growth hormones, siderophore, and toxins).^39^ The growth stimulation by *Trichoderma* is not plant-species dependent and has been ascribed to its positive effect on several regulatory enzymes (e.g., succinate dehydrogenase, glucose-6-phosphate dehydrogenase) of the plants’ citric acid cycle (energy generation) and the hexose monophosphate pathways (biosynthesis and cellular protection).^40^ Additionally, *Trichoderma* often leads a driving role in promoting crop yields^41^ by increasing nutrients’ availability and organic matter turnover within the soil through mechanisms such as changing the soil pH value, utilizing and recycling of organic into inorganic nutrients (thanks to the secretion of enzymes such as cellulases, glucanases, chitinases, proteinases, and xylanases), and improving availability of nutrients (e.g., iron and P solubilization through the secretion of siderophore and phosphatase).^21,42,43^ *Trichoderma* can further have a biostimulants effect through the synthesis of bioactive molecules (e.g., indole-3-acetic acid, ethylene, indole-3-ethanol, and indole-3-acetaldehyde that have auxin-like activity and promote the root development) and the release of secondary metabolites (e.g., 6-*n*-pentyl-6*H*-pyran-2-one, viridin, and siderophores) within the rhizosphere.^44,45^ All these biostimulants concur to increase nutrients bioavailability and their uptake by the roots, improve the communications between root and aerial plant structures,^21,45–47^ and boost root development and architecture, also affecting the exudation of carbohydrates to the advantage of fungal development.^48–50^ Additionally, *Trichoderma* can further lead to increased photosynthesis,^48,51^ with the upregulation of genes related to light-harvesting components, chlorophylls, and carotenoids,^52^ especially in tomatoes.^53,54^

The synergy between digestate and *Trichoderma* is essential to exploit the nutritional potential of the digestate and the biostimulant potential of both digestate and *Trichoderma* to the fullest and achieve comparable results to CF achieving circular economy and cost reduction goals.

### 3.2 Nutritional Results

Nutritional parameters of tomato fruits were evaluated, and the results are reported in Table 1. The DF + FI treatment presented the highest lycopene content (97.4 ± 1 mg kg^–1^ FW), while the lycopene content for DF + AGD was significantly lower (91.6 ± 4 mg kg^–1^ FW). Both CF (84.2 ± 0 mg kg^–1^ FW) and DF (77.4 ± 0 mg kg^–1^ FW) treatments showed lower lycopene content than bioactivated treatments but still higher than the NF control (56.7 ± 5 mg kg^–1^ FW) (Table 1). These results seemed to indicate that both fertilization and the presence of *Trichoderma* played roles in stimulating lycopene production. Fertilization and the consequent nutrients’ availability improved the lycopene content, i.e., DF presented a significantly higher lycopene content than NF, and the CF was further higher than DF. Therefore, nutrient availability affected both crop yield and lycopene content so well indicated by the correlation found between these two parameters (*r* = 0.89; *p* < 0.01; *n* = 3). Additionally, the presence of *Trichoderma*, independently by nutrient availability, determined a further increase in the lycopene content, so that bioactivated DF treatments presented the highest lycopene content (Table 1). These results highlight the efficacy of *Trichoderma* to promote the lycopene biosynthesis, such as previously reported.^14,53^ The biosynthesis of lycopene in plants and its regulation has been reported to be related to genetics, healthy status, environmental stimuli, and interactions with microorganisms.^55,56^ In particular, the lycopene biosynthesis is also regulated by several phytohormones, like abscisic acid (ABA), ethylene (ET), and jasmonate, that were reported to be released by *Trichoderma* spp.^51^ and enhance the lycopene synthesis into the tomato fruits.^56–58^

The TSS concentration is an important parameter that determines the sweetness of tomatoes, and it is used as a reference for the quality of tomatoes and their use in the food sector. The TSS content in fruits is strictly determined by variety, ripening stage, and conservation.^59^ Results indicated that fertilization affected the TSS content. All digestate’s treatments showed higher TSS content (*P* < 0.05) (DF: 6.4 ± 0.1 °Bx; DF + FI: 6.8 ± 0.1 °Bx; DF + AGD: 6.5 ± 0.1 °Bx) than those indicated for both CF (5.3 ± 0.0 °Bx) and NF (5.5 ± 0.1 °Bx) treatments, the presence of *Trichoderma* seemed to improve the TSS content (e.g., DF + FI) (Table 1).

The combination of increased TSS and lycopene might derive by the mechanistic process highlighted by Coppola et al.;^60^ the high TSS observed may result from the overexpression of genes linked to metabolic and cellular processes, often seen with *Trichoderma* spp., these plant sugars and the increased expression of glycolytic enzymes could further redirect sugars within tomato into biosynthetic pathways for resistance-related secondary metabolites, consistent with the upregulation of terpene and carotenoid (e.g., lycopene) genes and consequent production observed in tomato plant treated with *Trichoderma*. These mechanisms were possibly enhanced when the consortium was added as a water suspension rather than in a granulated format.

Different fertilization treatments also affected the macronutrient content in the tomato: CF had the highest N content (17.3 ± 0.1 mg kg^–1^ DW), while the N content ranged from 12.1 to 13.8 mg kg^–1^ DW (*P* < 0.05) within the other treatments. The similarity of N content between all organic treatments and the NF treatment highlights that the N-availability was higher in the CF treatment. Assuming that a higher N content in CF leads to a higher protein content,^61,62^ N availability could explain, also, the lower sugar content of CF, due to resource allocation and sugar trade-off, with respect the treatments fertilized with the digestate.^63^ Again, differences appeared for K, Ca, and in part Cu, for which activated digestate treatments always showed lower content than chemical treatment. These results were in contrast with previous studies^14^ that reported higher content for Mg, Cu, and Ca when the digestate was bioactivated because of the effect of *Trichoderma* in promoting Ca but above all K nutrition, significant differences between bioactivated and CF were observed.

### 3.3 Microbial Communities

When considering the designing of new formulations, especially for novel microbially activated fertilizers, two main components must be included, (i) the active ingredient (biocontrol agents, natural extracts, chemical molecules), in this case *Trichoderma*, and (ii) an auxiliary substance or excipient (inert substances that protect and aid the release of the formulation), in this case digestate.^64^ This second component can exert multiple functions such as ensure the product’s stability, protect the active ingredient, promoting adhesion to the target, retaining moisture, preventing desiccation, enhancing product dispersion, and facilitating application.^65^ The final goal of these formulations and combinations of the two components must ensure the maintenance of the microorganism in a state of low or inactive metabolism, while preserving its viability and effectiveness for the longest possible duration during storage.^66^ Within this study, the combination of FI and digestate’s solid fraction was tested within a granulated product since it offers enhanced environmental and user protection as the granular structure makes them less susceptible to absorbing moisture from the environment, which reduces water activity, minimizes contamination, and supports better survival of microorganisms.^67^ Additionally, the formulation within the digestate itself could further help *Trichoderma* development in the first stages, creating a niche rich in nutrients and biostimulant molecules to speed up the first developmental stages.

The effectiveness of a novel formulation must be then characterized at an environmental level, both biotic, to ensure its effects on the resident rhizospheric, plant and soil community, and its effects on the crop; and abiotic, to assess variations and interactions in soil properties and other agricultural variables but also at a management level in terms of added value, accessibility (e.g., versatility, adaptability, shelf life; protocols, guidelines, and regulations).^64^

Within this study, a further microbiological characterization was carried out to understand the impact of the new formulation over FI permanence and impact on the rhizospheric community.

#### 3.3.1 Permanence of the Inoculum

When the presence/absence of the FI was monitored across time, *Thrichoderma* was retrieved in the FI samples at an average abundance of 99.9%, as expected (Figure 2). Within the granules (ADG), the abundance of *Thrichoderma* was 38% (with the remaining percentage occupied by the community of the digestate). Both soil and digestate at the beginning of the experiment did not show the presence of the inoculated *Trichoderma*.

**Figure 2:**
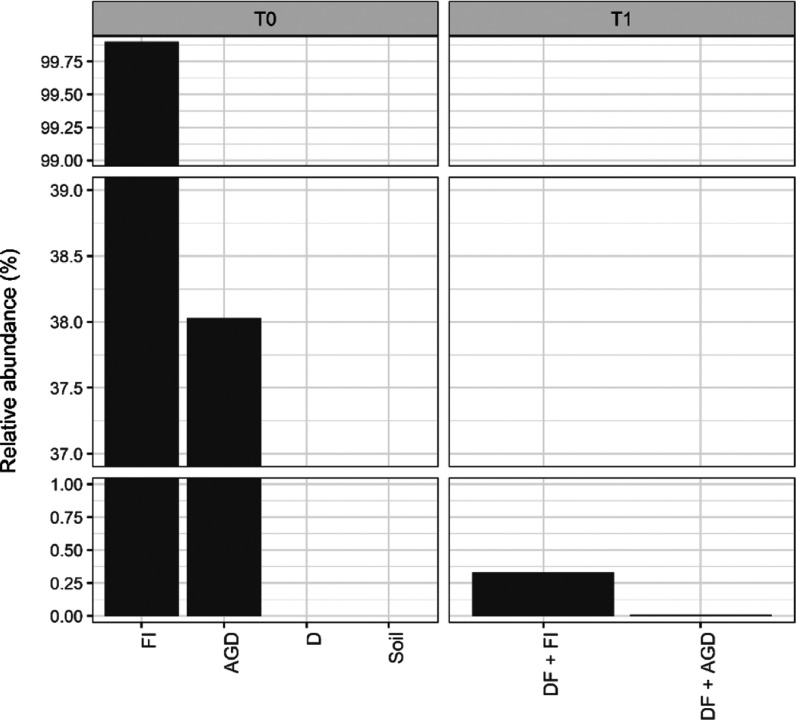
Bar-plots of the consortia microorganisms’ relative abundances across treatments and time (av.; *n* = 2).

When looking at the rhizospheric communities of the inoculated treatments, at the end of the experiment, *Thrichoderma* was retrieved in both DF + FI (0.3%) and DF + AGD (0.01%), confirming the permanence and acclimation of *Thrichoderma* within the rhizosphere but also a decrease in abundance from DF + FI to DF + AGD even when using the same dosage of FI.

In the case of filamentous fungi such as *Trichoderma*, an appropriate substrate is crucial. Low-cost organic materials, such as digestate, are preferred as they offer practical advantages for solid-state fermentation processes.^68^ The retrieved decreasing trend of *Trichoderma* abundances from DF + FI to DF + AGD could be due to several reasons that might require more in-depth future investigations such as (i) an artifact due to the experimental procedures also possibly due to the presence of humic substances and colloids in the digestate; (ii) the process of granulation that could have slightly impacted the fitness of *Trichoderma*; this hypothesis is however most likely to be excluded due to the suitable operational conditions of the palettization in terms of temperature and vacuum; (iii) the encapsulation itself that could have led to again a reduction of the vitality of *Trichoderma* by either exerting toxicity (likely to be excluded as seen in Section 3.1. and 3.2.) or hindering/delaying its release from the granules therefore reducing or pushing back *Trichoderma*’s growth. This slight reduction in *Trichoderma* abundance, however, seemed not to have affected yield results, which remained similar between the two treatments with a slight increase in DF + AGD but possibly could have an influence more on quality parameters such as lycopene and TSS.

#### 3.3.2 Rhizospheric Communities

In general, the sequencing produced between 112,874 and 296,686 reads after trimming and assembling and removal of the chimeras’ reads were between 89,410 and 233,056 ASVs (Table S4).

When looking at both the rhizosphere community, there was an increasing trend in richness for bacteria while in richness, evenness, and alpha-diversity for fungi with respect to T0, most likely due to tomato transplant and growth (Table S5).

In terms of beta diversity, the two theses treated with *Trichoderma* (DF + ADG and DF + FI) showed a different fungal rhizospheric community than the untreated one (*p* < 0.01) (Figure 3), a LEfSe analyses revealed an enrichment mainly in *Conocybe* (increase: +29,450%; final relative abundance: 6 × 10^–3^%), GS13 (+268%; final relative abundance 4 × 10^–5^%), and *Agaricostilbomycetes* (final relative abundance 2 × 10^–5^%) that were absent in the untreated soil (Figure S1).

**Figure 3:**
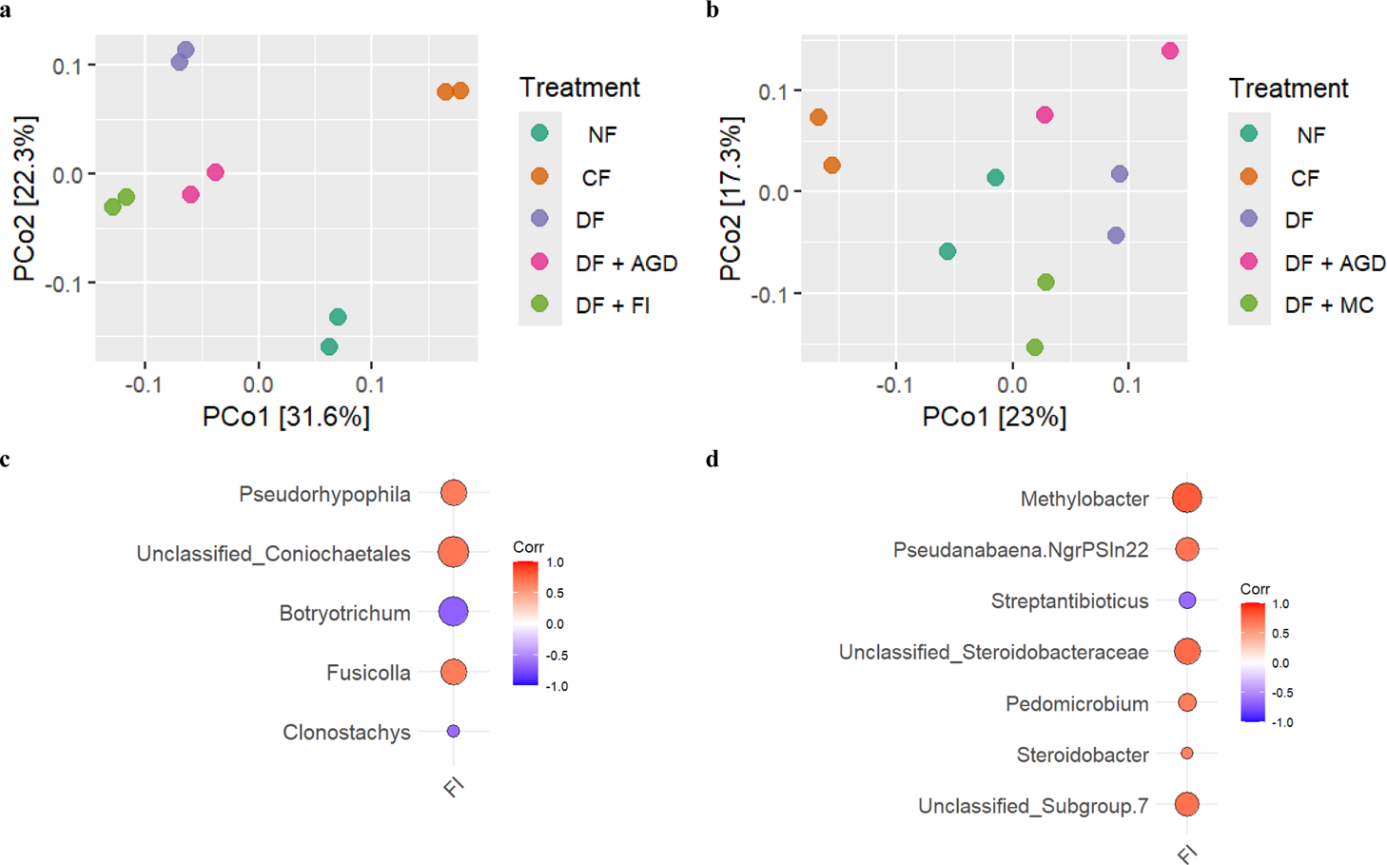
PCoA of the fungal (a) and bacterial (b) rhizospheric communities; co-occurrence for the statistically significant interactions (*p*-value <0.05) based on the Spearman rank correlation index of the inoculated *Trichoderma* against the most abundant (>0.5% in at least one sample) fungal (c) and bacterial (d) genera within the rhizospheric communities.

On the other hand, when looking at the bacterial community, a similar trend to the fungal communities could be seen between treated and untreated groups (Figure 3). The LEfSe analyses revealed an enrichment in *Aeribacillus* (+235%; 1 × 10^–4^%), microorganisms mainly known to degrading lignocellulose by secreting hydrolases,^69^ for samples treated with *Trichoderma* (Figure S1). The increase in lignocellulosic degraders hints to a higher degradation potential of soil organic matter, possibly linked also to digestate degradation, and the consequent release of a higher amount of organic molecules and nutrients then available for further degradation of *Trichoderma* and plant nutrition, further stimulating biosynthesis within tomatoes (e.g., TSS and lycopene).

A co-occurrence analyses revealed that the inoculated *Trichoderma* created 5 correlations (3 positive and 2 negative; *p* < 0.05) with fungal genera (above 0.5%), while 7 (6 positive and 1 negative; *p* < 0.05) with bacterial genera (above 0.5%) (Figure 3).

The fungal positive correlations were with *Fusicolla* (relative abundance in the inoculated samples: 0.01%), an unclassified *Coniochaetales* (0.005%) and *Pseudorhypophila* (0.007%), while the negative with *Botryotrichum* (0.01%), a genus known to contain plant pathogenic species,^70^ and *Clonostachys* (0.02%). *Fusicolla* is known to have antifungal activity and biocontrol mechanisms^71^ and has already shown positive interactions with *Trichoderma*.^72^ Similarly, strains of the *Pseudorhypophila* have been shown to possess antifungal activities^73^ while *Clonostachys* has been found with an ambivalent activity of both an antagonistic and beneficial fungi.^74,75^ *Trichoderma* species most often produce both volatile and nonvolatile compounds (e.g., antibiotics, terpenoids, steroids, and hydrolytic enzymes) that inhibit the growth of plant pathogens and prevent their colonization of host plants preserving plant health.^76,77^ *Trichoderma* is therefore known to compete with fungal pathogens and other soil fungi for vital resources; however, it has shown both positive and negative interactions with other plant-promoting fungi.^78^ The positive co-occurrence with other fungi with similar antipathogenic and plant stimulant activity found within this study might create a natural collaborative consortium amplifying *Trichoderma* indirect effects through mechanisms like promotion of plant and root development. Additionally, the understanding of the compatibility of *Trichoderma* with other fungal strains, and microorganisms in general, is essential to develop, customize, and optimize future synthetic microbial consortia and biobased fertilizers to improve plant health and productivity.^78^

The bacterial positive correlations were with *Methylobacter* (0.004%), *Pseudanabaena* NgrPSin22 (0.001%), an unclassified *Steroidobacteraceae* (0.001%), *Pedomicrobium* (0.004%), *Steroidobacter* (0.004%), and an unclassified subgroup 7 while the negative with *Strepantibioticus* (0.0005%), a relatively new genera known for producing antibiotics.^79^ *Methylobacter* has shown to create synergistic effect with mycorrhizal fungi,^80^ while *Pseudanabaena* has shown biostimulant activity.^81,82^ *Pedomicrobium* has already been encountered in other plant biostimulation studies possibly forming relationships with beneficial fungi; in particular unlike the other microorganisms retrieved within this study *Pedomicrobium* has been already previously found to positively correlate with *Trichoderma* biofertilization.^83,84^ Similarly, *Steroidobacter* has been found connected to improved plant stress resistance.^85^ *Trichoderma* often develops mutually beneficial partnerships that enhance plant and soil health. Adaptive mechanisms such as cell wall modification and ROS metabolism enable *Trichoderma* to selectively associate with compatible bacteria, shifting from antagonism to cooperation. Cooperative interactions between these microbes can strengthen plant immunity and improve biocontrol against pathogens often contributing to nutrient cycling, stress tolerance, and agroecosystem stability, offering sustainable alternatives to chemical control.^86^

Summarizing, *Trichoderma* showed a high persistence in tomato rhizosphere indicating a driving beneficial role in the microbial activation of digestate, and simultaneously *Trichoderma* seemed to establish multiple positive interactions with other beneficial rhizospheric microorganisms, thereby fostering synergy and amplifying their beneficial effects. Exploring microorganisms found to positively correlate with *Trichoderma* could further pave the way to new synthetic consortia centered around *Trichoderma*.

Overall, the microbially AGD allowed to overcome the low nutrient availability of digestate’s solid fraction, leading to tomato yield not significantly different from the one obtained by using chemical fertilizers. Moreover, fruit quality was improved by bioactivated organic treatments, which elicited a higher lycopene content than the chemical treatment. These results appear of great importance in a frame of circular economy promoting a more sustainable agriculture, where materials with low nutrient values, i.e., the solid fraction of digestate, are upgraded to fertilizers thanks to bioactivation. These novel formulations have the potential to reduce the use of synthetic mineral fertilizers that promote biological and organic fertilization. In addition, the full-scale approach of this work (TRL 8) suggested the feasibility in producing such biofertilizers, paving the way to future business prospects.

## Supplementary Material



## Data Availability

The data sets generated during and/or analyzed during the current study are available in the NCBI SRA repository (BioProject accession number: PRJNA1256514) [https://www.ncbi.nlm.nih.gov/bioproject/ PRJNA1256514].

## Notes

Organic matrixes characterization, soil characterization, fertilization scheme, number of reads retained at each step of the taxonomic assignment through DADA2, observed richness, diversity indexes and evenness, and LEfSe of the fungal and bacterial rhizospheric communities (PDF)
