# Supplementary material for: Trichoderma-Activated Granulated Digestate as an Alternative to Chemical Fertilization: Effects on Tomato Yield and Quality, and Soil Rhizospheric Communities
Source: ACS Agric Sci Technol. 2025 Nov 21;5(12):2383–92. doi: 10.1021/acsagscitech.5c00338 (PMC12710862; doi:10.1021/acsagscitech.5c00338)

Supplementary materials for

Trichoderma-activated granulated digestate as an alternative to chemical fertilization: effects on tomato yield and quality, and soil rhizospheric communities.

Tihomir Petrov Petrov[a,#], Mattia Rizzetto[a,#], Elisa Clagnan[a]*, Marta Dell'Orto[a], Patrizia De Nisi[a], Giuliana D'Imporzano[a], Marco Ovani[b], Marco Pierpaolo Pina[b], Roberto Kron-Morelli[c], Fabrtizio Adani[a]

Affiliations:

[a] Gruppo Ricicla labs., Dipartimento di Scienze Agrarie e Ambientali – Produzione, Territorio, Agroenergia (DiSAA), Università degli studi di Milano, Via Celoria 2, 20133 Milano, Italy.

[b] Wrote Srl, Via Plinio, 1, 20124 Milano, MI, Italy.

[c] Agrifutur Srl, Via Campagnole 8, 25020 Alfanello, BS, Italy.

[#] Contributed equally.

***Corresponding author:** elisa.clagnan@unimi.it

Summary

Supplementary tables Page 2

Table S1. Organic matrixes characterization (mean ± std. dv.; n=3). Page 3

Table S2. Soil characterization (mean ± std. dv.; n=3). Page 4

Table S3. Fertilization scheme. Page 5

Table S4. Number of reads retained at each step of the taxonomic assignment through DADA2 (av. ± st. dev.; n=2). Page 6

Table S5. Observed richness, diversity indexes and evenness (av. ± st. dev.; n=2). Page 7

Supplementary figures Page 8

Figure S1. LEfSe of the fungal (a) and bacterial (b) rhizospheric communities. Page 8

Supplementary tables

Table S1. Organic matrixes characterization (mean ± std. dv.; n=3).

	D			AGD		
**Dry weight** (% FW)	26.83	±	3.04	46.23	±	0.06
**Volatile solids** (% DW)	85.02	±	0.36	76.44	±	1.18
**TOC** (% DW)	44.23	±	3.71	44.48	±	4.05
**TKN** (g/kg DW)	24.41	±	1.79	24.38	±	1.63
**$N-NH_4$** ($g\ kg^{-1}$ FW)	1.94	±	0.01	1.95	±	0.04
**P** (g/kg DW)	7.91	±	0.08	16.41	±	1.21
**K** (g/kg DW)	12.78	±	0.40	28.17	±	2.39
**pH** (pH unit)	8.9	±	0.0	8.7	±	0.0

Table S2. Soil characterization (mean ± std. dv.; n=3).

	Soil		
**Skeleton > 2 mm** (g kg^{-1} DW)	1	±	1
**Sand 2-0.05 mm** (g kg^{-1} DW)	317	±	8
**Silty 0.05-0.002 mm** (g kg^{-1} DW)	536	±	13
**Clay < 0.002 mm** (g kg^{-1} DW)	146	±	6
**pH H_2O** (pH unit)	7	±	0
**pH $CaCl_2$** (pH unit)	6	±	0
**$CaCO_3$** (g kg^{-1} DW)	< 5		
**TC** (g kg^{-1} DW)	10.8	±	0.1
**TOC** (g kg^{-1} DW)	10.5	±	0.1
**OM** (g kg^{-1} DW)	18.1	±	0.2
**TN** (g kg^{-1} DW)	1.2	±	0.0
**C/N ratio**	8.6	±	0.1
**Total acidity** (pH 8.2) (meq 100 g^{-1} DW)	8.2	±	0.1
**Ca exchange** (mg kg^{-1} DW)	1,585	±	30
**Mg exchange** (mg kg^{-1} DW)	178	±	3
**K exchange** (mg kg^{-1} DW)	121	±	5
**Na exchange** (mg kg^{-1} DW)	33.4	±	1.6
**Potential CEC** (meq 100 g^{-1} DW)	18.1	±	0.0
**Ca/Mg ratio**	5.3	±	0.2
**Mg/K ratio**	4.8	±	0.3
**ESP** (%)	0.8	±	0.0
**Assimilable P** (mg kg^{-1} DW)	70.5	±	0.7

Table S3. Fertilization scheme.

	**N**	**P**	**K**
**Plant requirements** (kg ha^{-1})	130 (N)	100 (P_2O_5)	185 kg ha^{-1} (K_2O)
**NF** (kg ha^{-1})	0	0	0
**CF** (kg ha^{-1})	282 (Urea)	165 ($Ca(H_2PO_4)_2$)	360 (K_2SO_4)
**DF** (kg$_{DW}$ ha^{-1})	5,327 (D)	5,327 (D)	5,327 (D) + 219 (K_2SO_4)
**DF+FI** (kg$_{DW}$ ha^{-1})	5,327 (D)	5,327 (D)	5,327 (D) + 219 (K_2SO_4)
**DF+AGD** (kg$_{DW}$ ha^{-1})	5,191 (D) + 136 (ADG)	55,191 (D) + 136 (ADG)	5,191 (D) + 136 (ADG) + 219 (K_2SO_4)

Table S4. Number of reads retained at each step of the taxonomic assignment through DADA2 (av. ± st. dev.; n=2).

		**Prokaryotic**						**Eukaryotic**					
		**Input**	**Filtered**	**Denoised Fw**	**Denoised Rv**	**Merged**	**Non chimeric**	**Input**	**Filtered**	**Denoised Fw**	**Denoised Rv**	**Merged**	**Non chimeric**
**T0**	**Soil**	209158 ± 5652	186067 ± 5521	180482 ± 5181	179964 ± 5148	166032 ± 4549	154800 ± 4201	209900 ± 5651	182987 ± 4733	181225 ± 4743	181078 ± 4811	177501 ± 4838	169625 ± 5068
	**D**	206735 ± 1069	183154 ± 901	180807 ± 901	180591 ± 809	171258 ± 2080	157023 ± 2524	156908 ± 44034	129332 ± 37788	128993 ± 37726	129005 ± 37724	127498 ± 36998	126163 ± 36753
	**FI**	260346 ± 13922	241695 ± 10651	239895 ± 10678	239441 ± 9840	175844 ± 36074	165570 ± 38874	207246 ± 4866	186172 ± 1427	186147 ± 1421	186128 ± 1449	185634 ± 1553	181809 ± 1139
	**ADG**	172581 ± 33996	154073 ± 29189	153423 ± 29332	153374 ± 29486	151067 ± 29985	140772 ± 32355	210051 ± 7388	188009 ± 5626	187832 ± 5630	187777 ± 5686	187072 ± 5437	183461 ± 5232
**T1**	**NF**	205373 ± 1286	183633 ± 3664	177129 ± 4838	176188 ± 5881	159303 ± 8946	146773 ± 10677	188642 ± 12164	163902 ± 7542	162059 ± 7678	161922 ± 7832	158161 ± 8174	152706 ± 8055
	**CF**	205369 ± 3293	181634 ± 71	176121 ± 200	175581 ± 385	161305 ± 123	150075 ± 69	212567 ± 7282	183565 ± 6434	181698 ± 6482	181568 ± 6428	177539 ± 6142	171445 ± 6527
	**DF**	209734 ± 7055	187943 ± 8756	182421 ± 8627	182257 ± 9383	167275 ± 9458	156489 ± 9324	205057 ± 66	168797 ± 1874	167070 ± 1885	166979 ± 1807	162902 ± 1687	157930 ± 1904
	**DF+FI**	206357 ± 370	186886 ± 5177	180536 ± 5211	178911 ± 5827	163067 ± 5571	150766 ± 6001	204822 ± 737	170109 ± 1420	168140 ± 1349	167900 ± 1240	163817 ± 656	157762 ± 172
	**DF+AGD**	250373 ± 46313	229022 ± 47391	222678 ± 46680	222450 ± 46728	205167 ± 43895	191480 ± 41576	223277 ± 28120	193012 ± 27713	190656 ± 27172	190503 ± 27177	185341 ± 25915	176613 ± 22967

Table S5. Observed richness, diversity indexes and evenness (av. ± st. dev.; n=2).

		Prokaryotic				Eukaryotic			
		**Observed**	**Shannon**	**Simpson**	**Evenness**	**Observed**	**Shannon**	**Simpson**	**Evenness**
**T0**	**Soil**	4188 ± 64 a	7.42 ± 0.03 a	1 ± 0.01 a	0.89 ± 0.01 a	840 ± 9 b	4.67 ± 0.01 b	0.99 ± 0.01 a	0.70 ± 0.01 a
	**D**	1101 ± 7 b	5.28 ± 0.03 b	0.99 ± 0.01 a	0.76 ± 0.01 ab	423 ± 41 c	3.63 ± 0.08 c	0.93 ± 0.01 b	0.61 ± 0.03 b
	**FI**	504 ± 50 b	3.78 ± 0.01 b	0.94 ± 0.01 a	0.61 ± 0.01 b	6 ± 1 d	0.16 ± 0.01 e	0.07 ± 0.01 d	0.09 ± 0.01 d
	**ADG**	1502 ± 584 b	4.86 ± 1.06 b	0.89 ± 0.11 a	0.67 ± 0.11 b	103 ± 6 d	1.29 ± 0.03 d	0.67 ± 0.01 c	0.28 ± 0.01 c
**T1**	**NF**	4371 ± 48 a	7.36 ± 0.01 a	1 ± 0.01 a	0.88 ± 0.01 a	1179 ± 6 a	5.1 ± 0.07 a	0.99 ± 0.01 a	0.73 ± 0.01 a
	**CF**	4326 ± 88 a	7.36 ± 0.01 a	1 ± 0.01 a	0.88 ± 0.01 a	1051 ± 49 ab	4.84 ± 0.04 ab	0.99 ± 0.01 a	0.70 ± 0.01 a
	**DF**	4369 ± 111 a	7.44 ± 0.02 a	1 ± 0.01 a	0.89 ± 0.01 a	996 ± 19 ab	4.91 ± 0.06 ab	0.99 ± 0.01 a	0.72 ± 0.01 a
	**DF+FI**	4459 ± 142 a	7.36 ± 0.03 a	1 ± 0.01 a	0.88 ± 0.01 a	1141 ± 88 a	5.09 ± 0.09 a	0.99 ± 0.01 a	0.73 ± 0.01 a
	**DF+AGD**	4653 ± 652 a	7.36 ± 0.1 a	1 ± 0.01 a	0.88 ± 0.01 a	1173 ± 92 a	5.08 ± 0.04 a	0.99 ± 0.01 a	0.72 ± 0.01 a

Supplementary figures

Figure S1. LEfSe of the fungal (a) and bacterial (b) rhizospheric communities.

a

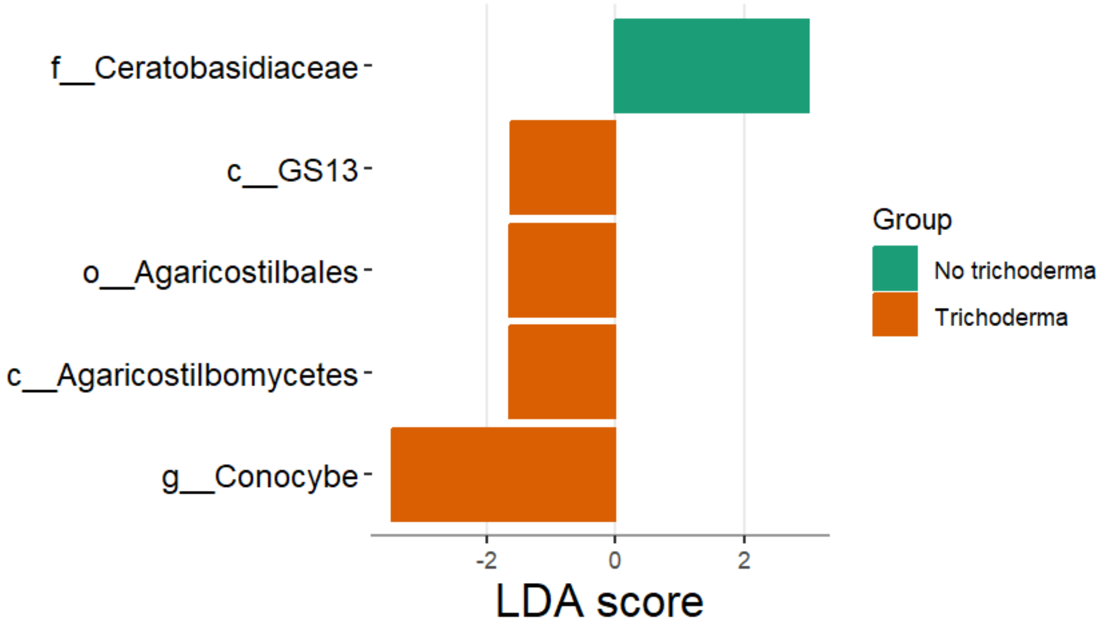

b

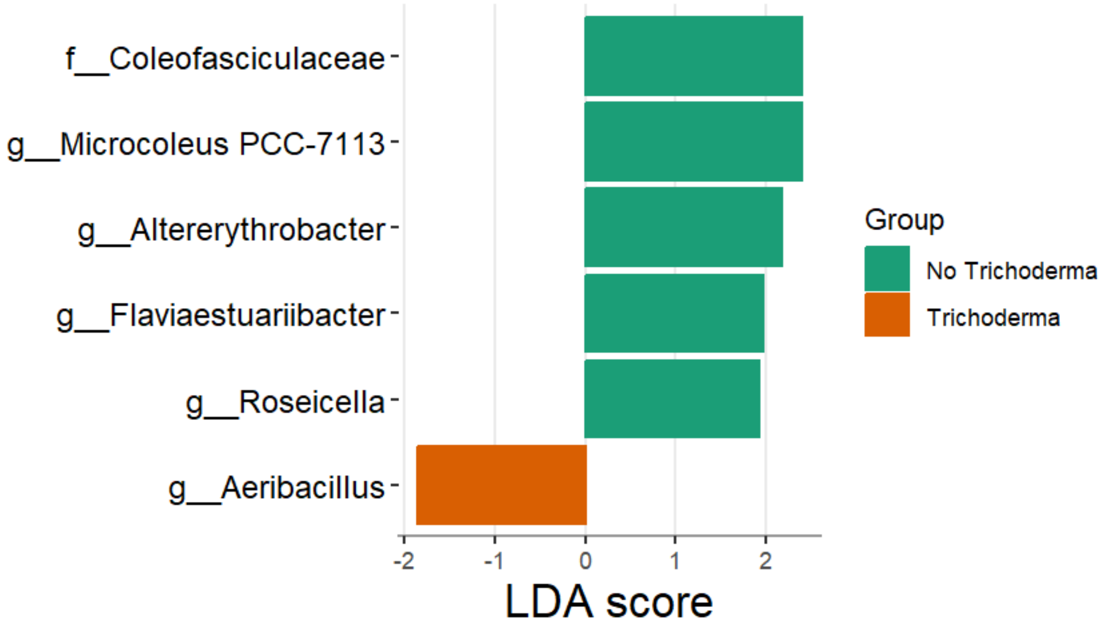

Supplement: Supplementary file 1 [file as5c00338_si_001.pdf]
